# Strategies adopted by oral physicians, oral and maxillofacial surgeons, and oral pathologists in patient education on oral cancer: A Nigerian study

**DOI:** 10.1002/cnr2.1929

**Published:** 2023-10-26

**Authors:** Kehinde K. Kanmodi, Afeez A. Salami, Adam A. Gbadamosi, Jacob N. Nwafor, Babatunde A. Amoo, Akinyele O. Adisa, Timothy O. Aladelusi, Bello Almu, Jimoh Amzat, Ramat O. Braimah, Mike E. Ogbeide, Chukwubuzor U. Okwuosa

**Affiliations:** ^1^ Faculty of Dentistry University of Puthisastra Phnom Penh Cambodia; ^2^ Campaign for Head and Neck Cancer Education (CHANCE) Programme, Cephas Health Research Initiative Inc Ibadan Nigeria; ^3^ School of Dentistry University of Rwanda Kigali Rwanda; ^4^ School of Health and Life Sciences Teesside University Middlesbrough UK; ^5^ Department of Oral and Maxillofacial Surgery University College Hospital Ibadan Nigeria; ^6^ Department of Dental Surgery Federal Medical Centre Bida Nigeria; ^7^ Division of Medicine Nottingham University Hospital NHS Trust Nottingham UK; ^8^ African Field Epidemiology Network Abuja Nigeria; ^9^ Department of Oral Pathology/Oral Medicine University College Hospital Ibadan Nigeria; ^10^ Department of Oral Pathology/Oral Medicine University of Ibadan Ibadan Nigeria; ^11^ Department of Oral and Maxillofacial Surgery University of Ibadan Ibadan Nigeria; ^12^ Department of Sociology Usmanu Danfodiyo University Sokoto Nigeria; ^13^ Department of Sociology University of Johannesburg Johannesburg South Africa; ^14^ Faculty of Dental Sciences Usmanu Danfodiyo University Sokoto Nigeria; ^15^ Department of Dental and Maxillofacial Surgery Usmanu Danfodiyo University Sokoto Nigeria; ^16^ Department of Oral Pathology and Oral Medicine University of Nigeria Teaching Hospital Ituku‐Ozalla Nigeria

**Keywords:** dental, education, Nigeria, oral cancer, patient

## Abstract

**Background:**

The burden of oral cancer in Nigeria is increasing. Different studies have shown how public education on oral cancer have increased knowledge of oral cancer across populations, however, it is not known if these practices are adopted by oral physicians, oral and maxillofacial surgeons, and oral pathologists in Nigeria.

**Aims:**

To investigate the patient oral cancer education strategies adopted by oral physicians, oral and maxillofacial surgeons, and oral pathologists in Nigeria.

**Methods:**

This study adopted an analytical cross‐sectional study design. This study surveyed practicing oral physicians, oral and maxillofacial surgeons, and oral pathologists in Nigeria. An e‐questionnaire was used for this study. The data were analyzed using the SPSS Version 20 software, and a *p*‐value of <.05 was used to determine the level of statistical significance.

**Results:**

The study's response rate was 46.6% (75/161). The 75 participants were from the six geopolitical zones in Nigeria responded to the survey questionnaire. Even though more than half (43/75, 57.3%) of the respondents have never received any training since their post‐bachelor's degree qualification on the strategies that can be used in educating patients on oral cancer, majority (54/75, 72.0%) of them knew at least one education strategy; also, the most known (36/54, 66.7%) and utilized (33/54, 61.3%) strategy among those respondents who were aware of patient education strategy was health talk. Only 38.7% (29/75) of the respondents reported that health learning materials (posters, leaflets, fliers, and flipcharts) are available in their clinics, all of which were in insufficient quantities. Also, 93.3% (70/75) of the respondents opined that it is worthwhile that dental clinics/hospitals in Nigeria invest in the provision of oral cancer learning materials for patient use. Inferential statistical analysis did not reveal any significant relationship between the respondents' characteristics and their awareness and practice on patient oral cancer education strategies.

**Conclusion:**

This study identified that many oral physicians, oral and maxillofacial surgeons, and oral pathologists in Nigeria lack the needed capacity to educate their patients on oral cancer. There is a need to strengthen their capacity by giving them training on patient oral cancer education strategies, and by providing them with good quality and enough teaching aids.

## 1 INTRODUCTION

Oral cancers are cancers involving the oral cavity and the oropharynx.^1^ Oral cancer is the most commonly occurring malignant neoplasm in the head and neck region, and it has persistently been one of the commonest cancers in the world.^2^, ^3^, ^4^, ^5^ Over 90% of oral cancers are squamous cell carcinoma.^6^ Within the past three decades, the global burden of oral cancer has increased by one‐fold, and the burden is notably worse in developing countries, including Nigeria.^7^ In 2017, the global incidence rate of oral cancer was 4.84 per 100 000 persons while the mortality rate was 2.42 per 100 000 persons.^7^ Oral cancer is the fifteenth most common cause of cancer‐related mortality worldwide, and Nigeria takes a share of this public health burden.^7^ Furthermore, men and disadvantaged population groups have been reported to be more likely to experience oral cancer than other comparative groups.^8^, ^9^ Overall, the victims of oral cancer often have a low five‐year survival rate, which is about 50%–60%.^10^, ^11^ This shows that oral cancer is a health problem of grave global health concern.

Oral cancer more commonly affects the tongue, and less commonly the floor of the mouth, cheek, gingiva and other structures in the oral cavity and oropharynx.^12^ There are several etiological/risk factors of oral cancer; however, the major ones are preventable, and they include tobacco use, harmful alcohol consumption and infection with carcinogenic human papillomavirus (HPV) strains.^13^

Patient education on oral cancer risk factors, oral cancer screening, oral cancer self‐examination, and HPV vaccination are laudable strategies for oral cancer prevention and control.^14^, ^15^ These educational interventions can promote awareness and knowledge on oral cancer, and may bring about an expansion in the population who presents to primary care with precancerous or early form of the disease; hence reducing the rate of advance‐stage clinical presentation of the disease.^16^

Dentists in Nigeria—particularly those who are specialists in oral medicine, oral and maxillofacial surgery, and oral pathology—have a very crucial responsibility in the prevention, early diagnosis, and treatment of oral cancer, and sometimes may be the first clinician to diagnose such cases during routine examination.^13^ Unfortunately, factors such as fear of diagnosis, poverty, and poor awareness, have precluded early diagnosis in most healthcare settings in Nigeria.^17^ Therefore, patients are mostly managed at advanced stages of disease through multimodal approach involving tumor ablation, chemotherapy, radiotherapy, or palliation.^18^ Hence, the use of evidence‐based educational strategies to improve public knowledge on oral cancer, especially by oral physicians, oral and maxillofacial surgeons, and oral pathologists, is very crucial.

Different studies have been conducted to show how oral cancer education strategies have increased public awareness and knowledge of oral cancer across populations^13^, ^19^, ^20^, ^21^; however, it is not known if these practices are adopted by oral physicians (who are also known as oral medicine specialists), oral and maxillofacial surgeons, and oral pathologists in Nigeria. Therefore, this study aims to investigate the oral cancer education strategies adopted by oral physicians, oral and maxillofacial surgeons, and oral pathologists in Nigeria.

## 2 METHODS

### 2.1 Study design

This research adopted an analytical cross‐sectional study design, and the reporting of this research was based on the STROBE (STrengthening the Reporting of Observational studies in Epidemiology) guidelines.^22^

### 2.2 Study population

As at the study period, there were a total of 15, 120, and 26 practicing oral physicians, oral and maxillofacial surgeons, and oral pathologists, respectively, in Nigeria. Hence, the total population of eligible participants (i.e., study population) was 161.

### 2.3 Study instrument

The instrument was an electronic questionnaire (Google Form) used for this study. The questionnaire was developed through three stages, as done in previous empirical studies.^23^, ^24^, ^25^ The first stage involved the development of the questionnaire content through review of studies on cancer and oral health education.^26^, ^27^, ^28^, ^29^, ^30^, ^31^ The second stage involved the face validity assessment of the questionnaire content by field/research experts in health education, clinical dentistry, and dental public health; at this stage, all relevant feedback obtained from these experts was used to revise the questionnaire. The third stage involved the conduct of a pilot study among fifteen practicing non‐specialist dental surgeons in Nigeria. The final version of the questionnaire had 3 sections: A, B, and C. Section A obtained information about the sociodemographic characteristics (age, gender, area of clinical specialization, etc.) of the participants. Section B obtained information about the participants' experience regarding their clinical management of oral cancer cases; this section contains questions like “*Have you had any specific training on oral cancer after qualifying as a dental surgeon?*”, “*Have you ever managed a patient with oral cancer?*”, “*What is the most common type of oral cancer in your centre?*”, etc. Section C obtained information about issues concerning the strategies used in the participants' facility for patient education on oral cancer education; this section contains questions like “*Which of the following ways do you think can be used to prevent oral cancer in Nigeria?*”, “*Do you know of any cancer education strategy?*”, “*Which oral cancer education strategies have you ever used?*”, etc.

### 2.4 Sample size

Being a small population group, a whole population (*n* = 161) sampling was considered for this study.^32^

### 2.5 Sampling technique

Volunteer sampling technique was the sampling technique used for this study.

### 2.6 Data collection

Collection of data was done between 31 March 2023 and 10 April, 2023. All practicing oral physicians, oral and maxillofacial surgeons, and oral pathologists in Nigeria were invited to participate in this study, and this was done through gatekeepers. The gatekeepers used for this study were executives of the Medical and Dental Consultants Association of Nigeria (MDCAN) and those of the national societies of these three dental specialties. The invitation contains an introductory message, a participant information sheet (PIS), and a link to the electronic questionnaire (Google Form). All potential participants were invited, by the gatekeepers, through email or WhatsApp/Telegram messaging services. All potential participants were informed about the study objectives and benefits through the PIS. Through the PIS, they were also informed that their participation was strictly voluntary, anonymous, and confidential. Being an anonymous study, they were also informed that filling and submitting their responses to the questionnaire implied that they consented to participate in the study. Only those that were willing to participate in the study filled and submitted their responses.

### 2.7 Data analysis

The collected data were analyzed using the Statistical Package for Social Sciences (SPSS) Version 20 software (IBM Corp, New York, NY, United States). Descriptive statistics of all variables were done and presented in this study. Bivariate analysis (Chi‐square and Fischer tests) and multivariate analysis (logistic regression analysis) of variables of interest were also done in this study but were not presented in this study as these inferential statistics did not generate any statistically significant result (a *p*‐value of <.05 was considered as statistically significant). Chi‐square and Fischer tests were used to test associations between two variables while logistic regression analysis was used to determine if a variable is a predictor of another variable.

### 2.8 Ethical considerations

Participation in this study was completely voluntary, anonymous, and informed. Ethical clearance to conduct this study was obtained from the Sokoto State Ministry of Health (Ref. No. SMH/1580/V.IV).

## 3 RESULTS

The response rate to this study was 46.6% (75/161). The majority of the respondents were males (80.0%), within the age range of 31–50 years (58.7%), practicing in a tertiary care facility (94.7%), working in public practice (94.7%), practicing in the geopolitical zones in the southern part of Nigeria (74.7%), and oral and maxillofacial surgeons (70.7%) (Table 1).

**TABLE 1:** Socio‐demographic and professional characteristics of the respondents.

Variable (*N* = 75)	Frequency	Percentage
Age group (Years) – Mean = 45.61, SD = 9.39		
31–40	22	29.3
41–50	22	29.3
51–60	11	14.7
61–70	6	8.0
No response	14	18.7
Gender		
Male	60	80.0
Female	14	18.7
Prefer not to say	1	1.3
Years of clinical practice (Mean = 17.08, SD = 8.19)		
0–10	13	17.3
11–20	21	28.0
21–30	10	13.3
31 & above	5	6.7
No response	26	34.7
Practice setting		
Primary care	1	1.3
Secondary care	2	2.7
Tertiary care	71	94.7
Others	1	1.3
Ownership of practice		
Public (Government)	71	94.7
Private	2	2.7
Military	1	1.3
No response	1	1.3
Location of practice		
North central	6	8.0
North East	3	4.0
North West	10	13.3
South East	17	22.7
South South	8	10.7
South West	31	41.3
Area of specialization		
Oral and maxillofacial surgery	53	70.7
Oral pathology/medicine	22	29.3

Abbreviations: N, total number of respondents; SD, standard deviation.

Only a few (9.3%) respondents have managed less than 11 oral cancer patients in their years of clinical practice. The commonest (52.0%) oral cancer type at the respondents' center was oral cancer affecting other parts of the oral cavity excluding the lip, oropharyngeal area, palate, and the tongue. Importantly, less than half (46.7%) of the respondents reported that they always discuss the possible risk factors of oral cancer with patients at risk of oral cancer (Table 2).

**TABLE 2:** Experience of respondents on oral cancer management.

Variable (*N* = 75)	Frequency	Percent
Number of oral cancer patients ever managed		
None	1	1.3
1–10	6	8.0
11–20	6	8.0
21–30	12	16.0
31–40	7	9.3
41–50	41	54.7
No response	2	2.7
Commonest type of oral cancer at respondent's center		
Cancer of the lip	5	6.7
Cancer of the oropharyngeal area	8	10.7
Cancer of the palate	11	14.7
Tongue cancer	12	16.0
Cancer of other parts of the oral cavity	39	52.0
Frequency of discussing possible risk factors of oral cancer with patients at risk of oral cancer		
Rarely	3	4.0
Sometimes	23	30.7
Usually	14	18.7
Always	35	46.7

Abbreviation: N, total number of respondents.

**FIGURE 1:**
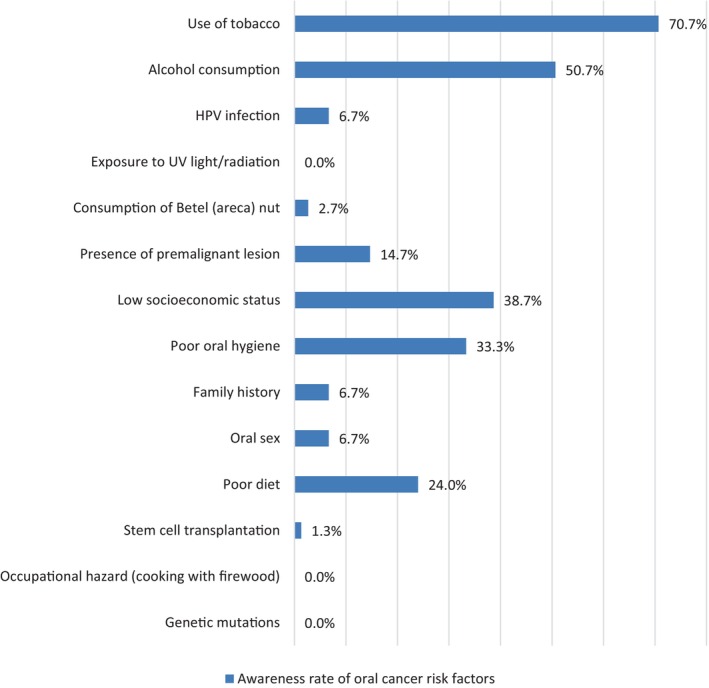
Knowledge of oral cancer risks among the respondents.

The three most known oral cancer risk factors among the respondents were tobacco use (70.7%), alcohol consumption (50.7%), and low socio‐economic status (38.7%) (Figure 1).

More than half (57.3%) of the respondents have never received any training on oral cancer education strategies since their post‐Bachelor of Dental Surgery degree qualification. Also, the majority (>90%) of them were aware of at least one oral cancer prevention strategy. However, of the four oral cancer prevention strategies asked from the respondents, oral cancer screening was the most known (94.7%) strategy among them (Table 3).

**TABLE 3:** Exposure to oral cancer prevention and oral cancer education strategies among the respondents.

Variable (*N* = 75)	Frequency	Percent
Received any post‐BDS training on oral cancer education strategies		
Yes	32	42.7
No	43	57.3
Oral cancer prevention strategies known among respondents^a^		
Oral cancer screening	71	94.7
HPV vaccination	51	68.0
Oral cancer self‐examination	51	68.0
Use of protective oral sexual barriers	30	40.0

Abbreviations: BDS, bachelor of dental surgery; N, total number of respondents.

^a^ Multiple responses allowed.

**FIGURE 2:**
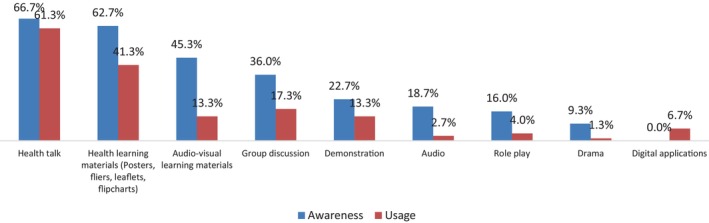
Awareness and usage of oral cancer education strategies among the respondents (*n* = 54).

Among those respondents (*n* = 54, 72.0%) that were aware of oral cancer education strategies, health talk, health learning materials (posters, leaflets, fliers, flipcharts), and audio‐visual learning materials were the most known and most used education strategy (Figure 2).

An overwhelming majority (93.3%) of the respondents opined that dental clinics/hospitals should invest in the provision of oral cancer learning materials for patient use. However, it is pertinent to note that only 38.7% of the respondents reported that they have health learning materials on oral cancer in their clinic.

Posters (79.3%), leaflets (41.4%), and fliers (34.5%), in descending order, were the top three most available health learning materials on oral cancer in the clinics of those respondents (*n* = 29) who reported the availability of health learning materials on oral cancer in their clinic. Most (79.3%) of these materials contained information on oral cancer risk factors while only very few (6.9%) contained information on the use of protective oral sexual barriers (dental dam, tongue condom, etc.). Importantly, majority (65.5%) of these respondents indicated that the quantities of these materials were not enough in their clinic while a few (10.3%) of them reported that those materials were of poor quality (Table 4).

**TABLE 4:** Status of health learning materials on oral cancer in the respondents' clinics.

Variable	Frequency	Percent
Opinion about the need for dental clinics/hospitals to invest in the provision of oral cancer learning materials for patient use (*n* = 75)		
Yes, it is worthwhile	70	93.3
No, it is not worthwhile	2	2.7
No response	3	4.0
Availability of health learning materials on oral cancer in the respondents' clinic (*n* = 75)		
Yes	29	38.7
No	31	41.3
Unsure	14	18.7
No response	1	1.3
Available health learning materials on oral cancer in the respondents' clinic^a^ (*n* = 29)		
Posters	23	79.3
Fliers	10	34.5
Leaflets	12	41.4
Use of digital applications	4	13.8
Flipcharts	1	3.4
Oral cancer/health issues addressed by health learning materials on oral cancer in the respondents' clinic^a^ (*n* = 29)		
Oral cancer risk factors	23	79.3
Oral cancer screening	22	75.9
Oral cancer self‐examination	14	48.3
HPV vaccination	9	31.0
Use of protective oral sexual barriers (dental dam, tongue condom, etc.)	2	6.9
Quantity of health learning materials on oral cancer available in the respondents' clinic (*n* = 29)		
Not enough	19	65.5
Enough but not in surplus	7	24.1
Surplus quantity	1	3.4
No response	2	6.9
Quality of health learning materials on oral cancer available in the respondents' clinic (*n* = 29)		
Poor quality	3	10.3
Good quality	24	82.8
Very good quality	1	3.4
Excellent quality	1	3.4

^a^ Multiple responses allowed.

## 4 DISCUSSION

The dearth of empirical evidence on the use of oral cancer education strategies by oral physicians, oral and maxillofacial surgeons, and oral pathologists in Nigeria to increase patient knowledge of oral cancer is apparent. Therefore, this survey was conducted to investigate the use of oral cancer education strategies by practicing oral physicians, oral and maxillofacial surgeons, and oral pathologists in Nigeria. The findings obtained in this survey were noteworthy and discussed below.

To start with, the response rate (46.6%) to this study was below average. This observation is similar to the low response rates recorded in some previous questionnaire‐based surveys conducted among healthcare professionals in other climes.^33^, ^34^ Also, the use of online questionnaire as the study instrument may be another factor contributing to the low response rate recorded in this study, as existing research evidence has shown that the use of online questionnaire is usually associated with a relatively lower response rate, compared to paper‐based questionnaire.^35^, ^36^, ^37^ Additionally, the non‐use of incentives to stimulate participation in this study may also be a contributing factor, as research has shown that dentists respond more to incentivized surveys.^40^, ^41^ However, regardless of the low response rate recorded in this study, this study is believed to be representative of the sampled population as almost half of the total number of practicing oral physicians, oral and maxillofacial surgeons, and oral pathologists in Nigeria participated in this study. Additionally, respondents were recruited from each of the six geopolitical zones in Nigeria. This demonstrates that our sample composition is fairly distributed across Nigeria.

Also, the majority of the study respondents were males, within the fourth to fifth decade of life, and were practicing in tertiary/public healthcare facilities in the southern part of the country (Table 1). This finding is consistent with the existing findings concerning the general socio‐demographic profile of Nigerian clinicians.^40^, ^41^, ^42^ In Nigeria, most tertiary healthcare facilities are in urban or semi‐urban areas, and many Nigerian healthcare workers prefer to work in these areas because of better working conditions, availability of social services, and medical supplies/equipment.^42^, ^43^ This is in consonance with reports on the clinical workforce of many sub‐Saharan African countries.^44^ It is also noteworthy that the current state of insecurity in Nigeria, which is more predominant in the northern part of Nigeria, may also contribute to this disproportionately high concentration of clinicians in the southern part of Nigeria.^41^ This disproportionate distribution of oral physicians, oral and maxillofacial surgeons, and oral pathologists in Nigeria—that is, their overconcentration in the southern part of Nigeria—may negatively affect the screening, diagnosis, and treatment of oral cancer in the northern part of Nigeria. Hence, this may be a major factor perpetuating the burden of oral cancer disease in Nigeria.^7^

Furthermore, the respondents in this study were knowledgeable of diverse oral cancer risk factors and prevention strategies.^45^, ^46^, ^47^ This observation is not surprising, considering their expert background. However, majority (57.3%) of them have never received any training, since their post‐Bachelor of Dental Surgery degree, about the strategies they can use in educating their patients about what oral cancer is, how to prevent the disease, and how to get it treated. This is a very crucial finding because having an expert knowledge of a disease does not automatically translate to having an adequate patient teaching proficiency on such disease.^48^ Therefore, this necessitates the need for adequate training of the oral physicians, oral and maxillofacial surgeons, and oral pathologists in Nigeria on patient education strategies on oral cancer. This can be achieved through the provision of continuing medical education for them through seminars, workshops, and symposia.^49^, ^50^ The use of continuing medical education has been found to be effective in improving the capacity of dentists in the delivery of patient‐targeted intervention programs^49^; hence, such approach can be adopted among these group of dental specialists in Nigeria.

Clinical dental specialty training in Nigeria is unstructured; this may be a major factor which explains why less than half of the study respondents did not received training, during their clinical residency program, on how they can educate patients about oral cancer. Regardless of this, it is intriguing to note that despite the reported lack of such training among the respondents, an overwhelming majority of them were aware of at least one oral cancer education strategy, and this is quite commendable. Furthermore, health talk was the most known and most utilized oral cancer education strategy among them (Figure 2). The popularity of this strategy among them may be because it is easy to use, it is in real time, and at little or no cost compared to the use of other teaching strategies.^51^, ^52^

Asides health talk, the use health learning materials (posters, leaflets, and flyers) is also another somewhat popular educational strategy among the respondents; however, only a minority of them admitted that they have access to these materials in their facilities (Table 4). This finding is similar to a report among Mongolian dentists where insufficient learning materials was recorded as a major barrier to provision of oral health education by dentists.^53^ This is a serious issue of public health concern; therefore, investment into these learning materials by the administrators of healthcare facilities is very important as provision of facilities for proper patient education is a key function of hospital administrators globally.^54^ Therefore, investment into these health learning materials remains fundamental, as the use of such materials has been proven to be effective in promoting pro‐health and preventive behavior against cancers.^55^, ^56^

It is also noteworthy that less than half (46.7%) of respondents reported that they always discuss the risk factors of oral cancer with patients at risk of developing the disease (Table 2). This finding is similar to that reported among a sample of dentists in Canada where 43% of them reported to have discussed oral cancer risk factors with their patients.^51^ Overall, this finding demonstrates the need for comprehensive patient education on oral cancer and healthy behavior, as patient‐centered education helps to improve patient's knowledge of the preventive and control measures concerning a disease condition.^57^, ^58^

This study has its limitations. Firstly, the response rate was 46.6%. This relatively low response rate may be because the participation in this study was unincentivized. The provision of monetary incentives or certificates as a reward for participation in a study improves participation.^38^ Unfortunately, the study investigators were not able to provide incentives due to the paucity of funds, as this study was self‐funded. Also, the majority of the target population practice in tertiary healthcare centers which typically have a high volume of patient influx; hence, they have very busy schedules which may have not allowed them to enroll in the study.^39^ Secondly, the investigators employed an online mode of data collection which may likely exclude a certain population of specialists especially those who were not social media compliant or internet savvy, for example, the older specialists.^59^ Thirdly, the majority of the study participants were specialists practicing in the southern part of the country; hence, there was very little participation from those in the northern part, although the majority of the health workforce population in Nigeria is in the southern part of Nigeria.^40^ Therefore, the generalizability of these findings should be treated with caution.

Although this study has its limitations, nonetheless, its strengths are conspicuous. Though different studies have been conducted to show how oral cancer education strategies have increased public awareness of oral cancer across different populations,^51^, ^60^ however, this study has provided insights into the scope of patient‐centered oral cancer education strategies adopted by oral pathologists, oral and maxillofacial surgeons and oral physicians in Nigeria, who are the major specialists responsible for the screening, diagnosis, and management of oral cancer in the country. Also, the findings obtained from this study are crucial and the implementation of the recommendations made from this study in the Nigerian healthcare settings will provide a better approach for patient‐focused oral cancer education which should subsequently reduce the burden of oral cancer in Nigeria.

## 5 CONCLUSION

Oral cancer education increases oral cancer awareness thereby reducing risks of late presentation and rate of advanced‐stage oral cancer. Therefore, the need for patient‐focused oral cancer education in Nigerian healthcare facilities cannot be over‐emphasized, and importantly, oral physicians, oral and maxillofacial surgeons, and oral pathologists play vital roles in achieving this due to their expertise. Notably, the use of various health learning materials to achieve patient‐focused oral cancer education in Nigeria by oral physicians, oral and maxillofacial surgeons, and oral pathologists has been unfortunately met with challenges such as unavailability, gross insufficiency, and low material quality. Therefore, investment into these health learning materials is highly crucial to promote knowledge of oral cancer among patients and early presentations at healthcare facilities. Finally, postgraduate training on patient education on oral cancer should be encouraged among oral physicians, oral and maxillofacial surgeons, and oral pathologists in Nigeria to improve their capacity in patient education on oral cancer, thereby empowering patients to make conscious decisions on the need to carry out regular self‐examination and undergo other preventive measures against oral cancer.

## AUTHOR CONTRIBUTIONS

**Kehinde K. Kanmodi:** Conceptualization (lead); data curation (supporting); formal analysis (supporting); funding acquisition (lead); investigation (supporting); methodology (lead); project administration (lead); resources (lead); software (supporting); supervision (lead); validation (supporting); visualization (lead); writing – original draft (lead); writing – review and editing (lead). **Afeez A. Salami:** Conceptualization (lead); data curation (lead); investigation (lead); methodology (equal); project administration (equal); resources (equal); validation (equal); visualization (equal); writing – original draft (lead); writing – review and editing (lead). **Adam A. Gbadamosi:** Conceptualization (lead); data curation (equal); investigation (lead); resources (equal); validation (equal); visualization (equal); writing – original draft (lead). **Jacob N. Nwafor:** Conceptualization (lead); data curation (lead); investigation (lead); methodology (equal); project administration (lead); resources (lead); software (lead); supervision (lead); validation (equal); writing – review and editing (equal). **Babatunde A. Amoo:** Formal analysis (lead); investigation (lead); methodology (lead); resources (supporting); software (lead); validation (lead); writing – original draft (lead). **Akinyele O. Adisa:** Data curation (lead); investigation (lead); project administration (lead); resources (supporting); supervision (supporting). **Timothy O. Aladelusi:** Data curation (equal); investigation (supporting); project administration (supporting); resources (supporting); validation (supporting). **Bello Almu:** Conceptualization (supporting); investigation (lead); methodology (supporting); project administration (lead); resources (lead). **Jimoh Amzat:** Resources (supporting); writing – original draft (supporting); writing – review and editing (equal). **Ramat O. Braimah:** Data curation (equal); investigation (supporting); project administration (supporting); resources (supporting). **Mike E. Ogbeide:** Data curation (equal); investigation (supporting); project administration (supporting); resources (supporting). **Chukwubuzor U. Okwuosa:** Data curation (supporting); investigation (supporting); resources (supporting).

## FUNDING INFORMATION

This study was self‐funded.

## CONFLICT OF INTEREST STATEMENT

The authors have stated explicitly that there are no conflicts of interest in connection with this article.

## ETHICS STATEMENT

Ethical clearance to conduct this study was obtained from the Sokoto State Ministry of Health (Ref. No. SMH/1580/V.IV). Participation in this study was completely voluntary, anonymous, and informed.

## TRANSPARENCY STATEMENT

The lead author and manuscript guarantor, Kehinde Kazeem Kanmodi, affirms that this manuscript is an honest, accurate, and transparent account of the study being reported; that no important aspects of the study have been omitted; and that any discrepancies from the study as planned (and, if relevant, registered) have been explained.

## Data Availability

Data sharing is not applicable to this article as no new data were created or analyzed in this study.
